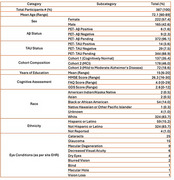# AI‐Based Hyperspectral Retinal Imaging for Alzheimer’s Disease Detection: Preliminary Insights from the Bio‐Hermes‐002 Study

**DOI:** 10.1002/alz70861_108584

**Published:** 2025-12-23

**Authors:** Sophie Grapentine, Alon Hazan, Tommaso Alterini, Anastasia Codirenzi, Justin DiGregorio, Sydney Hitchon, Tannis Kemp, Yi Ping Lin, Bar Mayo, Nir Oren, Anthony Rinaldi, Eliav Shaked, Catherine C Bornbaum, Ryan Visee, Mailis Bietenhader, Jean‐Michel Boudreau, Rozana Naureen, Joshua Weeks, Xuan Li

**Affiliations:** ^1^ RetiSpec, Toronto, ON Canada

## Abstract

**Background:**

Artificial intelligence (AI)‐based retinal hyperspectral imaging (rHSI) represents a promising, non‐invasive approach to detecting Alzheimer’s disease (AD)‐related pathology, offering scalability and potential point‐of‐care utility. Prior findings from Bio‐Hermes‐001 demonstrated strong concordance between RetiSpec’s rHSI model and amyloid PET (Aβ‐PET). Bio‐Hermes‐002 builds on this work in a racially and clinically diverse cohort. While full rHSI performance data are forthcoming, we report preliminary participant characteristics relevant to real‐world deployment of retinal imaging technologies.

**Method:**

Bio‐Hermes‐002 is an ongoing prospective, multi‐site study of ∼1,200 participants (*n* =387enrolled at time of reporting) aged 60‐89 (mean 72.1), stratified into cognitively normal, MCI, and mild‐to‐moderate AD cohorts. Retinal imaging was performed using RetiSpec’s rHSI system. Amyloid and Tau PET imaging is ongoing; thus, analyses to date are descriptive. Comparative analysis with PET will be presented.

**Result:**

Among enrolled participants, 57.4% were female and the cohort was both racially and ethnically diverse, with 18% of participants identifying as Hispanic or Latino and 19.4% identifying as non‐Caucasian. Notably, 19.4% of participants self‐reported eye conditions (e.g., n=25 cataracts, n=15 glaucoma, n=9 age‐related macular degeneration). Ophthalmologist review of images to confirm prevalence of comorbid eye conditions is forthcoming. The prevalence and distribution of eye conditions emphasizes the importance of validating retinal imaging tools in populations reflective of real‐world clinical settings, where such comorbidities are common.

Feasibility assessments suggest that high‐quality retinal images were obtainable across a broad range of participants, including those with eye conditions (e.g., cataracts), supporting the applicability of AI‐based rHSI in heterogeneous clinical populations. Analysis of retinal image quality and its relationship to ocular health status will be presented alongside AI model performance data relative to gold standard PET scans.

**Conclusion:**

Preliminary findings from Bio‐Hermes‐002 reinforce the feasibility of AI‐based retinal imaging in diverse and heterogeneous populations. Given the prevalence of ocular comorbidities such as cataracts, validation of retinal biomarkers under real‐world conditions is critical. Final results, including RetiSpec’s AI model performance against amyloid PET and comparative ensemble analyses, will be presented at the conference.